# Rhinitis in the Geriatric Population: Epidemiological and Cytological Aspects

**DOI:** 10.3390/geriatrics10020050

**Published:** 2025-03-20

**Authors:** Matteo Gelardi, Rossana Giancaspro, Elisa Boni, Mario Di Gioacchino, Giulia Cintoli, Michele Cassano, Maria Teresa Ventura

**Affiliations:** 1 Unit of Otolaryngology, Department of Clinical and Experimental Medicine, University of Foggia, 71121 Foggia, Italy; matteo.gelardi@unifg.it (M.G.); g.cintoli@hotmail.it (G.C.); michele.cassano@unifg.it (M.C.); 2 Allergy and Immunology Department, Metropolitan Laboratory of Bologna, 40133 Bologna, Italy; boni6055@gmail.com; 3 Institute of Clinical Immunotherapy and Advanced Biological Treatments, 66100 Pescara, Italy; digioacchino@me.com; 4 Department of Interdisciplinary Medicine, University of Bari, 70121 Bari, Italy; mariateresa.ventura@uniba.it

**Keywords:** allergic rhinitis, nasal cytology, geriatric age

## Abstract

Allergic rhinitis (AR), traditionally considered as a childhood condition, is increasingly recognized among older adults, driven by rising life expectancy and environmental factors. Although allergic sensitization declines with age, AR prevalence in the elderly is underestimated, with 3–12% of geriatric patients affected. Diagnosis is challenging due to nonspecific symptoms and overlapping conditions, leading to underdiagnosis and inadequate treatment. AR significantly impacts the quality of life (QoL), often exacerbating respiratory comorbidities like asthma and COPD. Presbynasalis, encompassing age-related sinonasal changes, includes reduced allergic responses, increased chronic rhinosinusitis, altered nasal structure, and impaired mucociliary clearance. Non-allergic rhinitis, atrophic rhinitis, and overlapping rhinitis further complicate AR diagnosis in the elderly. Effective management involves personalized pharmacotherapy, allergen-specific immunotherapy (AIT), and addressing comorbidities and polypharmacy risks. Despite safety concerns, recent studies demonstrate AIT efficacy in elderly patients, reducing symptoms and medication use. Given AR’s impact on cognitive and respiratory health, accurate diagnosis and treatment can enhance QoL and mitigate health decline. Greater awareness and further research are essential to understand AR prevalence and improve outcomes for geriatric patients.

## 1. Introduction

In recent decades, the increase in life expectancy has led to a progressive aging of the general population. Although this represents a great success for medicine and for the improvement of socio-economic conditions, it requires a critical review of the diagnostic and therapeutic approach to many pathologies. In fact, aging not only favors the onset of chronic-degenerative diseases, but also modifies the body’s response to traditional treatments, requiring tailor-made therapeutic strategies [1].

In this context, among the conditions that deserve particular attention, rhinopaties represent an emblematic example. In the elderly, nasal pathologies, often considered minor or transitory disorders, acquire increasing importance, as they significantly compromise patients’ quality of life. The reduction in mucociliary clearance, the alterations in the vascularization of the nasal mucosa, and the greater susceptibility to inflammatory and allergic processes are just some of the characteristics that make rhinopaties in the elderly an increasingly relevant field of study. Furthermore, comorbidities, the use of drugs that influence nasal function, and the greater fragility of the mucosa make a targeted therapeutic approach necessary, which takes into account the different age groups. Therefore, the reconsideration of rhinopaties is not limited to the simple management of symptoms, but extends to the search for new diagnostic and therapeutic solutions, with a view to personalized and multidisciplinary medicine.

## 2. Epidemiological Aspects

To guarantee so-called “successful aging”, it is essential to implement specific scientific support aimed at the growing social problems related to the aging of the population in Europe and, in general, in the world. As a matter of fact, epidemiological data increasingly highlight the need for healthy aging, in order to stem the growing economic burden deriving from the care of an increasing number of people over 65 in a “fragile” condition.

In this context, it is important to highlight that recent reports have indicated that the incidence of asthma and allergic rhinitis (AR), which are mostly regarded as high-burden diseases of childhood, may be underestimated in the elderly population. Indeed, although allergic sensitization is generally known to decrease with age, the increasing number of patients with allergies and the aging world population are causing an increase in allergic diseases in patients over 60 years of age [2]. As proof of this, the prevalence of Japanese cedar pollinosis in people in their 60s increased from 21.8% in 2008 to 36.9% in 2019 [3].

Hence the importance of focusing on the problem of allergic rhinitis in the elderly population, both to understand the influence of AR on their quality of life (QoL) and to define the needs of affected elderly people.

To date, only a few studies in the literature concern the evaluation of the epidemiology of AR in geriatric age, since the occurrence and natural course of allergic disease in elderly patients have been somewhat neglected. Recent studies suggest that between 3 and 12% and 4 and 16% of elderly patients suffer from AR and asthma, respectively [4]. However, these data are likely underestimated, as atopic diseases are often overlooked in older patients, due to the more challenging diagnosis in older than in younger individuals, requiring the exclusion of various other conditions that can cause similar nonspecific symptoms [5]. Moreover, most epidemiological studies have analyzed the epidemiological situation of rhinitis in adults or elderly people without considering the allergic mechanism [6]. As a result, AR in the geriatric age group has remained poorly perceived, so much so that a significant percentage of geriatric patients do not receive effective treatment [7].

It is worth noting the strong association between asthma and rhinitis, since about 80% of elderly subjects diagnosed with asthma have rhinitis, and among elderly subjects with rhinitis, 30% had asthma. The strength of this association increases very significantly with longer persistence and higher severity of rhinitis. These results confirm that an extensive nose–lung interaction is also present in the elderly, and data might also indicate a relationship between rhinitis and other respiratory diseases such as COPD, further underlining the importance of treating nasal diseases to improve the control of lung diseases [8].

## 3. Presbynasalis

Senescence is known to be characterized by lower sensitization to allergens and prevalence of allergic diseases compared to children and younger individuals. However, the prevalence of allergic rhinitis in older adults has been rising, as a consequence of both the increase in life expectancy and of climate change associated with air pollution [9,10].

The term presbynasalis encompasses all the changes that occur in the sinonasal tract caused by the aging process [11]. Key changes include a reduced allergic response, a higher frequency of chronic rhinosinusitis with nasal polyposis (CRSwNP) and rhinorrhea, diminished support for the external nasal tip, an increase in the internal nasal cavity volume, and a decline in the sense of smell.

In particular, aging is responsible for showing a marked decrease in ciliary beat frequency, a greater degree of microtubule disorganization, and prolonged nasal mucociliary clearance time, which in turn contribute, though not solely, to the higher rates of upper respiratory infections in elderly populations [12]. Moreover, both the weakening of the nasal epithelial barrier and the relevant significant changes in the immune response could determine an increase not only in the incidence of CRSwNP but also in the vulnerability to allergens and pathogens.

Senescence is also characterized by increased nasal resistance, decreased elasticity of nasal mucosa, an altered nasal cycle, and loss of external nasal support [13].

Other important aspects of presbynasalis are rhinorrhea, due to the increase in glandular activity and in viscosity of secretions, and smell disorders, which can also occur during the earliest stages of several neurologic disorders [14].

Distinguishing symptoms related to senescence from those associated with allergic diathesis therefore requires greater attention towards AR in the elderly, considering the negative impact that this disease in turn can have on the respiratory tract.

## 4. Clinical Features of Allergic Rhinitis

AR is an inflammatory disorder of the nasal mucosa caused by exposure to allergens, which triggers IgE-mediated inflammation [15]. Traditionally, AR has been classified according to the timing of allergen exposure, as seasonal, when symptoms occur in a specific season, or perennial, when symptoms persist throughout the year. However, this classification has limitations, as it can be difficult to clearly distinguish between seasonal and perennial symptoms. Over the years, the pollen season has been starting earlier and lasting longer. Moreover, nasal inflammation can persist for weeks after pollen exposure in patients with seasonal rhinitis, and most patients are sensitized to both pollen and perennial allergens [16].

To address these challenges, the Allergic Rhinitis and its Impact on Asthma (ARIA) Guidelines have introduced a new classification. Thus, AR is now categorized as intermittent when symptoms occur fewer than 4 days per week or for less than 4 consecutive weeks, and persistent when symptoms are present more than 4 days per week and for over 4 consecutive weeks [17].

Furthermore, symptoms are considered mild if they do not interfere with sleep or daily activities, and moderate/severe if they significantly impact sleep or daily functioning, or are otherwise bothersome.

**Figure 1:**
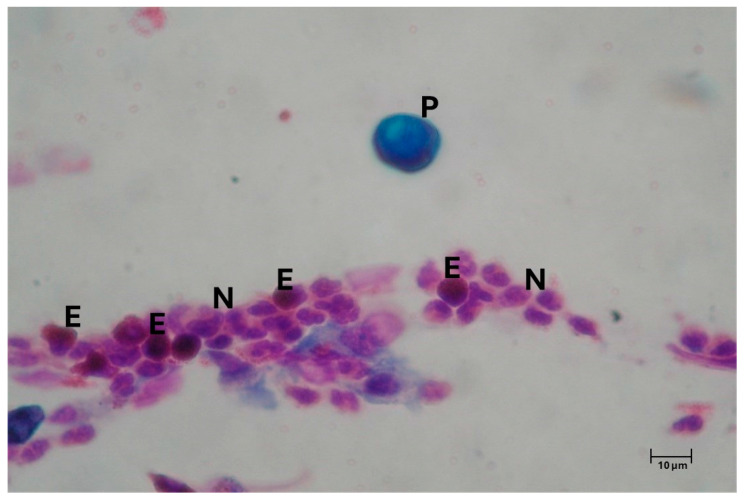
Nasal cytology of AR. MGG staining. Magnification 1000×. A rhinocytogram showing massive eosinophilic (E) and neutrophilic (N) inflammation. In the upper part of the image, an un-degranulated pollen grain (P) can be observed.

Patients with AR, whether naturally exposed or tested through specific nasal provocation tests, experience an allergic reaction with an early phase, primarily driven by histamine, followed by a late phase caused by inflammatory cells. From a cytological point of view, these responses are marked by the infiltration of inflammatory cells in the nasal mucosa, including eosinophils, mast cells, neutrophils, and plasma cells, which release various chemical mediators responsible for typical AR symptoms such as itching, nasal congestion, runny nose, conjunctivitis, and sneezing (Figure 1) [18]. Atypical symptoms are photophobia, ocular burning, and conjunctival dryness.

Nasal cytology findings from patients with perennial AR who are exposed to low-intensity but persistent allergens show “minimal persistent inflammation”, characterized by a continuous presence of neutrophils and, to a lesser extent, eosinophils. Conversely, rhinocytograms of seasonal AR patients differ based on allergen exposure. During pollen season, these patients exhibit all clinical signs and symptoms of AR, with NC showing degranulated neutrophils, lymphocytes, eosinophils, and mast cells. Outside of pollen season, however, both clinical symptoms and cytological findings are absent, showing a state of clinical and cytological silence [19].

In elderly people, Dermatophagoides pteronyssinus and Dermatophagoides farinae are considered the most common identified allergens [7]. Notably, given the age-related reduction in the production of IgE, atopy could not be defined by total IgE concentration but rather by means of specific IgE concentrations toward aeroallergens and/or skin prick test (SPT) positivity to at least one aeroallergen. The important clinical aspect is that, in older adults with relatively higher levels of IgE, atopic disease can be found in a higher prevalence and the severity of allergic conditions may be increased in older age, compared to younger people [20].

## 5. Other Rhinitis in Elderly People

### 5.1. Non-Allergic Rhinitis

Non-allergic rhinitis (NAR), also defined as “cellular rhinitis”, represents approximately 15% of all rhinopaties and is usually characterized by intense pseudo-allergic symptoms, which is why it is often confused with IgE-mediated rhinitis. According to NC findings, NAR can be classified as non-allergic rhinitis with neutrophils (NARNE), non-allergic rhinitis with eosinophils (NARES), non-allergic rhinitis with mast cells (NARMA), and non-allergic rhinitis with eosinophils and mast cells (NARESMA) [21].

**Figure 2:**
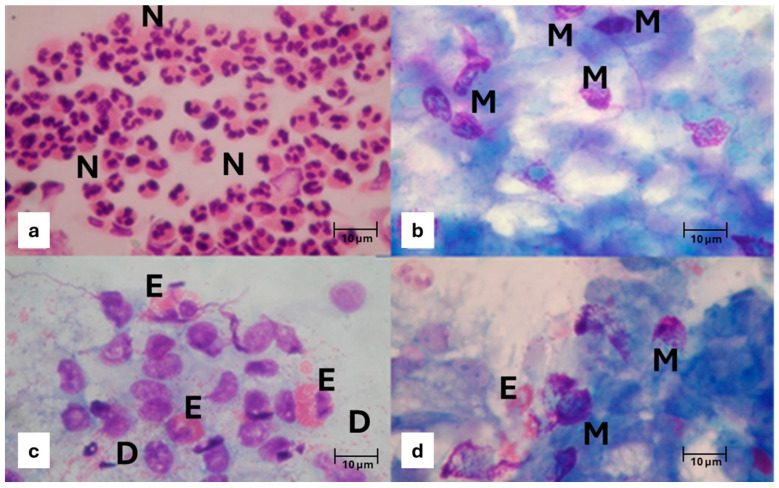
Nasal cytology of NAR. MGG staining. Magnification ×1000. (**a**) NARNE. N = Neutrophil. (**b**) NARMA. M = Mast cell. (**c**) NARES. E = Eosinophil; D = Degranulation. (**d**) NARESMA. E = Eosinophil; M = Mast cell.

NARNE is defined by a high presence of neutrophils (over 30%), without any detectable bacteria or fungal spores or hyphae (Figure 2a). The occurrence of this form of rhinitis has risen in recent years, probably due to factors such as industrialization and increased smoking rates, as it is more commonly observed among industrial workers, smokers, and people living in highly industrialized areas [22]. Gastroesophageal reflux is another contributing factor to NARNE, as hydrochloric acid expelled during exhalation can come into contact with the nasal mucosa, drawing neutrophils to this area [23]. The persistence of neutrophils and the release of inflammatory mediators, including neutrophilic elastase, leads to free radical formation and mucosal damage, resulting in symptoms such as sneezing, nasal congestion, and seromucous rhinorrhea [24]. NARMA is marked by the presence of partially degranulated mast cells in NC samples and can also be linked with systemic mastocytosis (Figure 2b) [25]. Symptoms related to this rhinitis type, such as nasal blockage, rhinorrhea, and sneezing, are usually intense. NARES is characterized by non-IgE-mediated nasal eosinophilic infiltration, often reaching 50–70% (Figure 2c) [26]. Eosinophils in the nasal mucosa may recruit mast cells through the production of various cytokines and chemokines, evolving into eosinophilic–mast cell rhinitis (NARESMA), which presents more frequent and severe symptoms (Figure 2d). This form of rhinitis is the most strongly associated with CRSwNP and asthma among the non-allergic rhinitis types and significantly affects patients’ quality of life (QoL) [27].

### 5.2. Overlapping Rhinitis

AR and NAR can coexist in the same patient in approximately 15–20% of cases. However, no studies have analyzed the incidence of overlapping rhinitis (OR) in the elderly population. Nevertheless, these overlapping forms of rhinitis present a significant clinical challenge, as patients with OR are frequently treated as if they had only allergic rhinitis, even when they experience severe symptoms outside of pollen seasons. In NC samples from these patients, eosinophils and/or mast cells are observed, even in the absence of exposure to specific allergens. Since therapeutic strategies specifically target allergic processes, patients with OR experience only partial improvement compared to their expectations. This highlights the importance of identifying, with the help of NC, the specific pathology affecting each patient, as only a correct diagnosis can ensure an effective treatment.

### 5.3. Iatrogenic Rhinitis

Iatrogenic rhinitis can be related both to the chronic use of nasal vasoconstrictors and to systemic drugs that also exert their action on the nasal mucosa, such as β-adrenergics, α-blockers, and oral ACE inhibitors. The latter drugs reduce sympathetic vascular tone, causing vasodilation and increasing nasal congestion [28,29].

This type of rhinitis is characterized by no variations in the cellular component at NC, unless concomitant allergic or infectious pathologies are associated [30].

### 5.4. Senile Rhinitis

Senile rhinitis is a clinical condition characterized by persistent, anterior watery rhinorrhea without a discernible trigger, often occurring in older adults. This condition is due to cholinergic hyper-reactivity, characterized by an exaggerated response of the nasal glands to the parasympathetic nervous system, leading to increased mucus production [31]. Gustatory rhinitis is one of the best examples of nasal hyperresponsiveness of the parasympathetic neural system after sensory nerve stimulation. Patients, in particular elderly ones, present with acute onset of annoying rhinorrhea, occurring immediately after the ingestion of liquid or solid foods [32,33].

**Figure 3:**
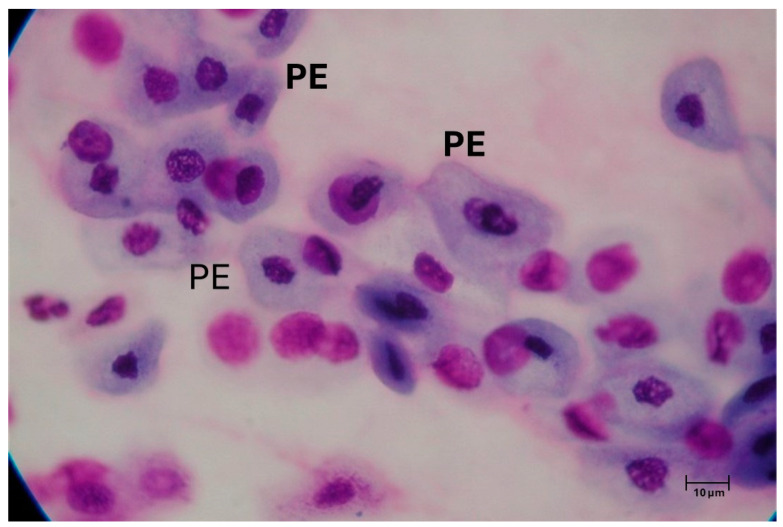
Nasal cytology of atrophic rhinitis. The mucous epithelium is represented by platicellular elements (PE) that have replaced the ciliated and mucous cells. MGG staining, 1000× magnification.

Elderly patients can also suffer from atrophic rhinitis, which is also associated with changes in the mucous epithelium, which undergoes progressive atrophy, with a reduction in goblet cells and an increase in squamous cells (Figure 3). This leads to nasal congestion, crusts formations, and cacosmia.

### 5.5. Chronic Rhinosinusitis

Chronic rhinosinusitis (CRS) is one of the most common inflammatory diseases, affecting about 5–15% of the general population in Europe and the USA. CRS is estimated to be the sixth most common chronic disease in the geriatric population [34]. 

## 6. Comorbidities

In elderly individuals, comorbidity plays a crucial role. Many people over 65 regularly use at least three to five different medications, leading to potential issues with drug interactions and challenges in setting treatment priorities. The complexity of these treatment regimens can also make it difficult to distinguish drug-induced symptoms from those caused by the underlying disease, especially in patients who may struggle to clearly communicate their symptoms or remember if they have followed the treatment plan accurately. Depression, a common condition (affecting roughly 39–40% of elderly patients), is frequently linked with anosmia, rhinitis, and sleep disturbances. Rhinitis itself can contribute to mental confusion and difficulties with spatial and temporal orientation. Meanwhile, medications used to treat depression can cause nasal dryness and rhinitis, making it challenging to pinpoint the source of these nasal symptoms [35,36].

Other prevalent comorbidities include gastroesophageal reflux disease (GERD) and obstructive sleep apnea (OSA). Although the connection is not fully understood, these conditions are likely associated with nasal inflammation, which could explain their link to rhinitis. The association between rhinitis and asthma also appears to increase with the severity of rhinitis, with a recent study indicating a particularly high prevalence of asthma in elderly patients with moderate-to-severe persistent rhinitis [8].

More uncommon conditions associated with rhinitis include sarcoidosis and amyloidosis.

## 7. The Treatment of Allergic Rhinitis in Elderly Patients

Elderly patients with allergic rhinitis (AR) are often undertreated compared to younger patients, and they also tend to have poorer responses to treatments. Several factors contribute to this, including immunosenescence, comorbidities, polypharmacy, mucosal atrophy, coexisting non-allergic rhinitis, and cognitive or adherence challenges in elderly patients [7,37].

The management of rhinitis in the elderly involves pharmacological treatment with both topical and oral medications, allergen-specific immunotherapy, and, when possible, avoidance of environmental allergens. Nasal irrigation may also help improve the nasal response to topical treatments. First-line medications typically include intranasal corticosteroids and intranasal or oral antihistamines. However, the elderly are often on multiple medications, increasing the risk of drug interactions and adverse effects, which are more common with polypharmacy. Furthermore, in the presence of hepatic or renal conditions, the risk of side effects rises, with anticholinergic drugs being particularly problematic, as they can cause urinary retention and delirium.

Among nasal corticosteroids, those like mometasone, budesonide, fluticasone, and beclomethasone are generally better tolerated and more effective than oral corticosteroids, with fewer undesirable effects such as hypertension or diabetes. However, they can cause side effects such as epistaxis, nasal dryness, and nasal burning. Oral corticosteroid use requires careful monitoring for adverse reactions [38,39].

Antihistamines must be carefully selected for elderly patients. First-generation antihistamines, due to their sedative effects, are often avoided, especially given the increased prevalence of glaucoma and prostate enlargement in older adults. Second-generation antihistamines, such as fexofenadine, cetirizine, and loratadine, are preferred as they lack sedative effects and have a safer profile. However, caution is necessary for certain antihistamines, such as mizolastine, which can affect cardiac repolarization, and rupatadine and ebastine, especially for patients with arrhythmias. Additionally, these newer antihistamines may interact with other drugs metabolized by the liver’s cytochrome P450 system, which can affect drug metabolism, especially in elderly patients with compromised liver or renal function [40].

The dose of some antihystamines should be adapted in patients with hepatic dysfunction and renal insufficiency. Bilastine should be considered a safe option in older patients since it has been proven to have no effect on the cholinergic receptor and no dose adjustment is required [41].

Decongestants are not typically first-line treatments for rhinitis but can be used cautiously in the elderly due to potential side effects, including increased blood pressure, headaches, and exacerbation of glaucoma or urinary issues [42].

Leukotriene inhibitors, such as cysteinyl leukotrienes (CysLT), are generally well tolerated by elderly patients and can enhance the treatment of seasonal allergic rhinitis. However, it is worth mentioning that older adults treated with leukotriene inhibitors may be particularly susceptible to anxiety and sleeping disorders [43,44].

Anti-IgE therapies, like omalizumab, are approved in some countries for severe allergic rhinitis, and studies confirm their safety in the elderly [45,46].

The use of allergen immunotherapy (AIT) in the elderly was largely overlooked until the last decade. Despite concerns over safety, particularly regarding severe reactions and potential interactions with medications like ACE inhibitors or beta-blockers, recent studies have demonstrated AIT’s efficacy and safety in elderly populations. Early studies have shown that AIT can be as effective in older adults as in younger individuals, with significant clinical improvements and reduced medication use [47]. More recent studies involving sublingual immunotherapy (SLIT) for dust mites and grass pollen allergies have shown positive outcomes, including reduced symptoms and less reliance on medication [48]. In the case of adverse reactions to AIT, newer medications like selective β-blockers and monoamine oxidase inhibitors do not significantly interfere with epinephrine treatment for anaphylaxis.

Thus, despite the challenges posed by comorbidities and polypharmacy, AIT is a viable and effective treatment option for elderly patients with allergic rhinitis, and cardiovascular diseases are not necessarily a contraindication to AIT.

## 8. Future Research Directions

Nasal cytology, which already allows us to identify immunophlogosis and distinguish the different types of rhinitis, will increasingly be associated with immunohistochemistry and immunofluorescence techniques. These advanced diagnostic methodologies will allow us to identify specific biomarkers of the disease, guaranteeing a more precise classification of rhinitis. This will favor the development of increasingly targeted treatments, improving the efficacy of therapies and reducing side effects, especially in elderly patients, who are often more vulnerable to generic and less personalized treatments.

The future of treatment, in fact, will include the approval of monoclonal antibodies, already used in other inflammatory diseases, which can be adapted to modulate specific immunological pathways involved in rhinitis. Furthermore, the use of probiotics and modulation of the nasal microbiota could emerge as an innovative therapeutic option to reduce chronic inflammation and improve the local immune response.

## 9. Conclusions

The diagnosis of AR in the elderly is more challenging because it is often misdiagnosed as senile rhinitis and also because of the coexistence of OR. In this context, nasal cytology and allergy tests appear of fundamental importance to ensure a tailor-made diagnosis and treatment.

When prescribing pharmacotherapy, it is essential to consider that older adults respond differently to drugs due to age-related organ changes, and they are also more susceptible to drug interactions due to the typically higher number of medications they take. Although aging itself does not rule out the use of AIT, it should be prescribed carefully in elderly patients who meet clear indications. Indeed, some conditions such as cancer and autoimmune diseases or even medications may contraindicate or complicate AIT. As a result, the risks and benefits of AIT need to be evaluated with particular care.

Nonetheless, AIT, particularly in the form of sublingual immunotherapy (SLIT), has been proven to substantially improve quality of life by alleviating symptoms and reducing the need for medication.

Since the impact of AR of elderly patients’ QoL is often more severe than in younger people, affecting cognitive function and potentially causing continual health decline, effective treatments could interrupt this cycle, enabling better symptom control with minimal reliance on symptomatic drugs, thus enhancing overall well-being for elderly patients. Further studies are needed to better define the prevalence of AR in elderly people, in order to raise awareness of this disease, thus reducing the risk of underdiagnosis and the negative impact on QoL.

## Data Availability

Data available on request from the authors.

## Abbreviations

AIT	Allergen immunotherapy
AR	Allergic rhinitis
CRS	Chronic rhinosinusitis
CRSwNP	Chronic rhinosinusitis with nasal polyps
GERD	Gastroesophageal reflux disease
NARES	Non-allergic rhinitis with eosinophils
NARESMA	Non-allergic rhinitis with eosinophils and mast cells
NARNE	Non-allergic rhinitis with neutrophils
OSA	Obstructive Sleep Apnea
